# Atypical presentation of secondary hepatic lymphoma with severe hepatic involvement: A case report and literature review

**DOI:** 10.1097/MD.0000000000043411

**Published:** 2025-07-18

**Authors:** Dongliang Zhao, Lai Wei, Lihong Li, Yanying Wang

**Affiliations:** a School of Clinical Medicine, Tsinghua University, Beijing, China; b Hepatopancreatobiliary Center, Beijing Tsinghua Changgung Hospital, Beijing, China; c Department of Hematology Oncology, Beijing Tsinghua Changgung Hospital, Beijing, China.

**Keywords:** diffuse large B-cell lymphoma, jaundice and abdominal pain, secondary hepatic lymphoma

## Abstract

**Rationale:**

While secondary hepatic lymphoma can develop in up to 50% of patients with non-Hodgkin lymphoma and approximately 20% of those with Hodgkin disease, it is uncommon for patients to present with severe hepatic involvement, manifesting initially as jaundice and abdominal pain. These nonspecific symptoms can complicate diagnosis and subsequently delay the initiation of appropriate treatment.

**Patient concerns:**

A 68-year-old female presented to our hospital with complaints of abdominal pain and jaundice.

**Diagnoses:**

Laboratory tests and clinical symptoms are often nonspecific for this atypical presentation of secondary hepatic lymphoma, and imaging findings can be challenging to distinguish. Histopathological examination remains the gold standard for diagnosis.

**Interventions:**

Due to severe thrombocytopenia and coagulopathy, only low-dose prephase chemotherapy was administered along with supportive treatments, including platelet transfusion and coagulation factor supplementation.

**Outcomes:**

The patient was dead. No autopsy was performed, as the patient’s family declined consent.

**Lessons:**

Clinicians should maintain high vigilance for atypical presentations of lymphoma, especially when encountering unexplained cholestatic hepatitis and cytopenias, and promptly initiate diagnostic evaluations to avoid delays in treatment. A definitive diagnosis primarily relies on histopathological examination, with chemotherapy remaining the cornerstone of treatment.

## 1. Introduction

Secondary hepatic lymphoma (SHL) refers to the involvement of lymphoma in the liver from an extrahepatic origin. It is more common than PHL, occurring in approximately half of non-Hodgkin lymphoma cases and in about one-fifth of Hodgkin lymphoma cases.^[1]^ SHL primarily affects middle-aged and elderly individuals, with a slightly higher incidence in men compared to women. Diffuse large B-cell lymphoma (DLBCL) is the most prevalent subtype. Diagnosis largely depends on imaging, which typically reveals multiple hepatic lesions and may show accompanying extrahepatic lymphadenopathy. However, histopathological analysis remains the gold standard for confirming SHL. Systemic chemotherapy is the primary treatment, with the specific regimen tailored to the lymphoma subtype. This paper aims to explore the challenges in diagnosing and treating SHL, particularly in cases lacking typical symptoms and imaging findings, with the goal of enhancing diagnostic and therapeutic efficiency for this condition.

This case report is based on retrospective analysis of clinical data and pathology obtained during routine patient management. Ethical approval was waived by the Institutional Review Board of Beijing Tsinghua Changgung Hospital due to the retrospective nature of the study and the absence of any additional experimental procedures beyond standard clinical practice. Written informed consent was obtained from the patient’s family members for publication of clinical information and accompanying images.

## 2. Case report

### 2.1. Case background

A 68-year-old woman, initially presented to our hospital 1 year ago with complaints of abdominal pain. Given her 20-year history of gallstones, an abdominal computed tomography (CT) scan at that time revealed a thickened gallbladder wall. No hepatic lesions were detected on imaging, and surgical intervention was recommended. However, the patient opted for conservative management. The abdominal pain persisted intermittently until January of this year, when it worsened, accompanied by fever (Tmax 38.6°C) and jaundice, leading to her hospitalization in the hepatobiliary surgery department. Due to the illness, the patient was emaciated in the past 3 months, with a weight loss of 20 kg.

Physical examination revealed a flat and soft abdomen without abdominal wall varicosities. The liver and spleen were nonpalpable, indicating no hepatomegaly or splenomegaly and Murphy’s sign was absent. There was no percussion tenderness or shifting dullness in the liver and kidney regions. Bowel sounds were normal, and no edema was observed in the lower extremities. Magnetic resonance cholangiopancreatography (MRCP), contrast-enhanced magnetic resonance imaging (MRI) of the upper abdomen, and contrast-enhanced CT scan of the upper abdomen showed no significant bile duct dilatation or pancreaticobiliary tumors. Consequently, conservative treatment was continued.

During hospitalization, thrombocytopenia, anemia and abnormal monocyte proportion were noted, prompting a transfer to the hematology department for further evaluation. The patient has a 40-year history of rheumatoid arthritis, complicated by secondary Sjögren’s syndrome for 10 years, for which she has been regularly treated with hydroxychloroquine and methotrexate. She underwent a hysterectomy for uterine fibroids 20 years ago. The patient has no history of smoking, alcohol use, or other significant illnesses.

### 2.2. Laboratory findings

Over the course of a year, the patient underwent periodic laboratory and imaging assessments to monitor her disease status. On November 10, 2023, she initially sought medical attention due to abdominal pain. At that time, apart from a slightly increased proportion of monocytes (MONO%: 11.3%, normal range: 5%–9%), no other significant abnormalities were found in the laboratory tests, imaging studies revealed no enlarged lymph nodes or hepatic masses.

**Figure 1.**
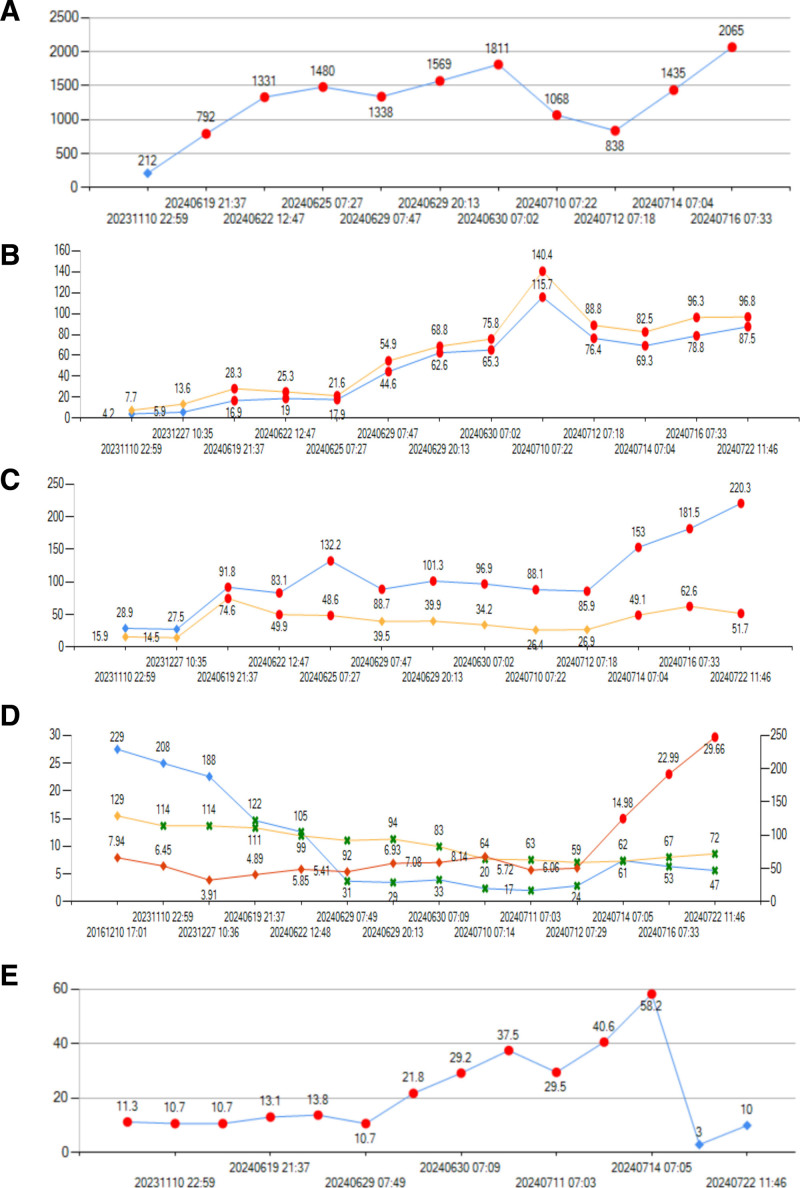
(A) Lactate dehydrogenase. (B) Orange represents total bilirubin and blue represents direct bilirubin. (C) Orange represents alanine transaminase and blue represents aspartate transaminase. (D) Orange represents hemoglobin, blue represents platelets, red represents white blood cells. (E) Proportion of monocyte.

By June 2024, the patient began experiencing persistent right upper abdominal distension and pain, accompanied by fever and weight loss. Although imaging studies still did not reveal any clear space occupying lesions, there was a marked worsening of anemia, a significant drop in platelet count, and the proportion of monocytes was continuously increasing. Additionally, transaminase and bilirubin levels steadily increased (Fig. 1).

**Figure 2.**
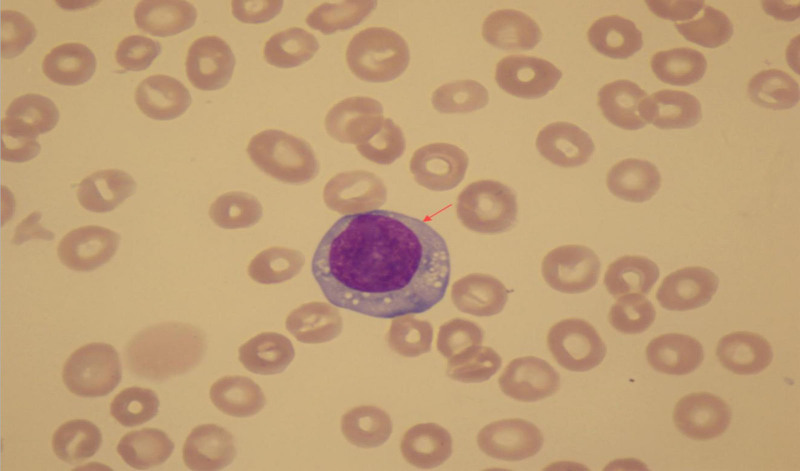
Under oil immersion (×100), the peripheral blood smear highlights an abnormal lymphocyte (indicated by the red arrow). These cells are larger than normal lymphocytes, exhibit loose chromatin, abundant gray-blue cytoplasm, and contain visible vacuoles.

**Figure 3.**
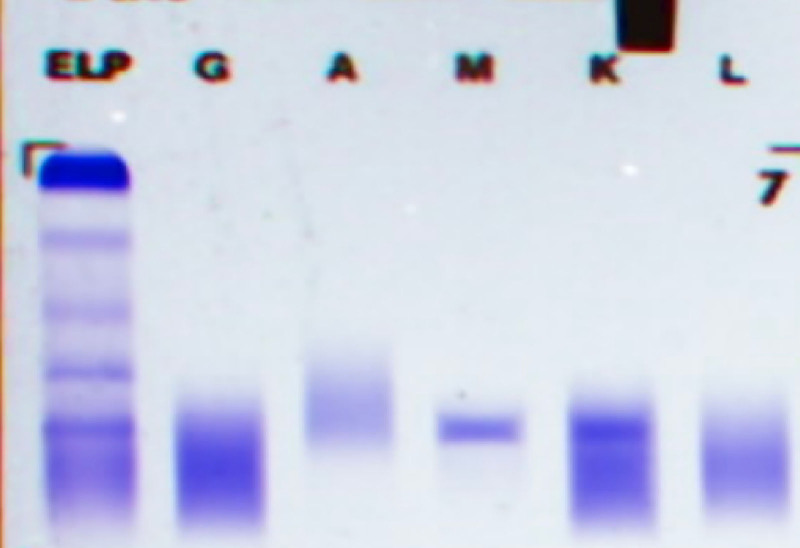
Serum immunofixation electrophoresis revealed an M-protein band, which formed a specific precipitation reaction with anti-IgM and anti-κ antibodies. IgM = immunoglobulin M.

Despite aggressive anti-infective treatment, the patient’s symptoms did not improve, and her overall condition continued to deteriorate. Further investigations revealed a continuous increase in the proportion of monocytes. A peripheral blood smear subsequently confirmed that these monocytes with an unusually high proportion were, in fact, abnormal lymphocytes (Fig. 2). Biochemical testing showed a slight elevation in immunoglobulin IgM, and immunofixation electrophoresis (IFE) confirmed the presence of monoclonal immunoglobulin IgM-kappa (Fig. 3).

Liver involvement was evidenced by elevated transaminase and bilirubin levels, hepatocellular jaundice, and a marked decline in synthetic liver function, reflected by abnormal coagulation parameters, decreased fibrinogen, and reduced albumin levels. Coagulation studies showed prothrombin time (PT) 16.2 seconds, activated partial thromboplastin time (APTT) 43.9 seconds, and INR 4.64, indicating significant coagulopathy. Bone marrow involvement manifested as severe thrombocytopenia and worsening anemia. The patient’s worsening condition was likely attributed to accelerated disease progression, with the rapid increase in lactate dehydrogenase (LDH) further supporting the assessment of aggressive tumor progression.

### 2.3. Bone marrow and cerebrospinal fluid examination findings

**Figure 4.**
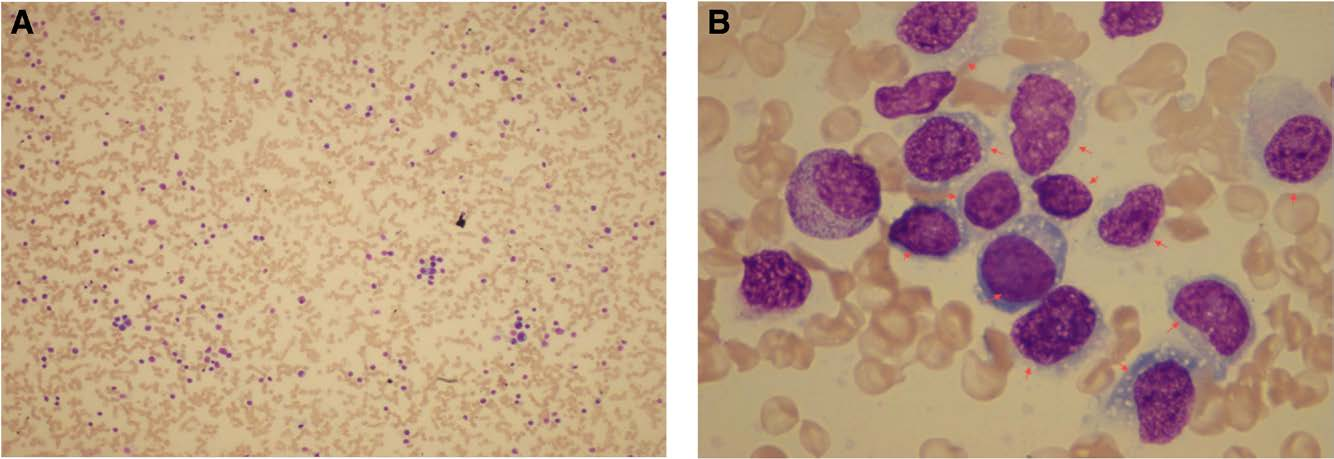
(A) Bone marrow smear observed under a low-power microscope (×10). (B) Bone marrow smear under oil immersion (×100), the red arrow indicates abnormal lymphocytes.

After abnormal lymphocytes were detected in the peripheral blood, bone marrow aspiration and biopsy were performed on the patient’s right iliac bone. The bone marrow smear revealed that 75% of the cells were abnormal monoclonal lymphocytes, similar to those observed in the peripheral blood. These cells varied in size, exhibited relatively abundant cytoplasm that was bright and dark blue, and contained visible vacuoles (Fig. 4).

Flow cytometry (FC) of the bone marrow demonstrated 47.84% monoclonal mature B lymphocytes (of all cells), which were positive for CD20, CD22, CD79b, HLA-DR, and BCL-2, but negative for CD5, CD10, CD23, CD38, CD43, CD200, CD25, CD11c, CD103, CD123, CD305, Ki-67, TdT, CD34, CD3, CD7, CD56, and CD33. Cytoplasmic Ig light chain Kappa showed restricted expression.

**Figure 5.**
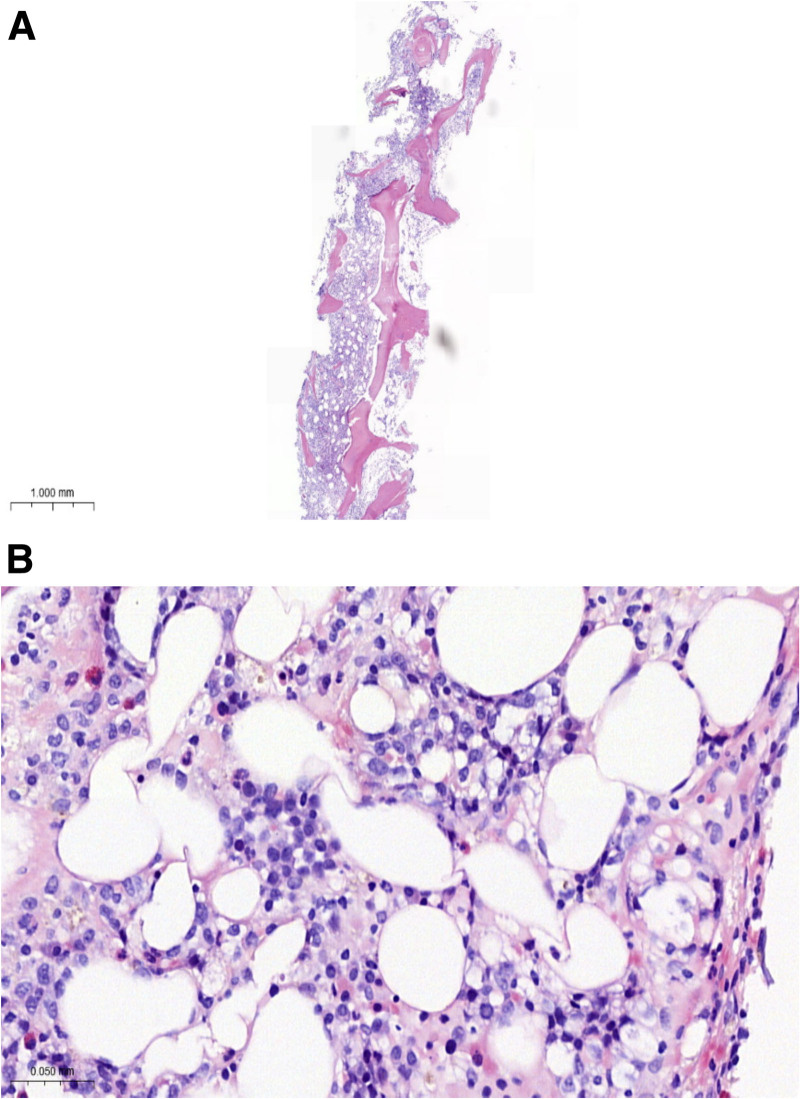
(A) Bone marrow biopsy pathology under a low-power microscope (×40). (B) Bone marrow biopsy pathology under a high-power microscope (×400).

Bone marrow biopsy revealed diffuse and sheet-like tumor cell infiltration within the interstitium. The tumor cells were relatively large, with vacuolated nuclei and visible nucleoli in some cells. Immunohistochemical analysis showed positivity for CD20, CD19, CD10, BCL-6 (60%), MUM1 (40%), BCL-2, C-MYC, and weak positivity for P53 (60%), while being negative for CD3, CD5, TdT, CyclinD1, CD23, and CD138. The Ki-67 labeling index was 75%. These findings confirmed B-cell lymphoma involving the bone marrow (Fig. 5).

A lumbar puncture was performed, and analysis of the cerebrospinal fluid revealed no malignant cells, confirming the absence of central nervous system involvement.

### 2.4. Imaging examination findings

**Figure 6.**
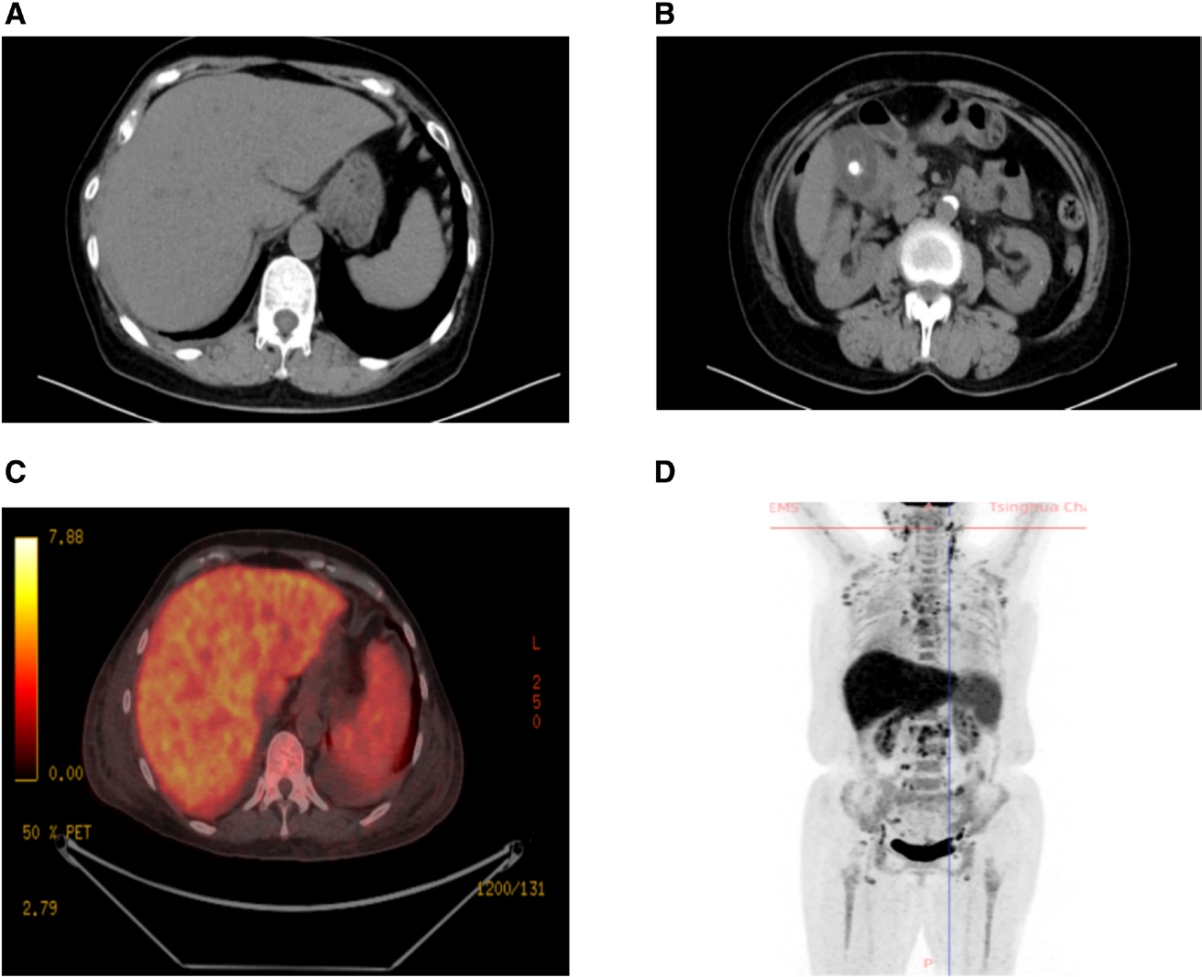
(A) No obvious liver space occupying lesions are seen in this computed tomography. (B) Gallbladder stones are visible in this image. (C) Significantly increased hepatic radioactivity uptake in this positron emission tomography. (D) Multiple lymph node enlargement accompanied by increased radioactivity uptake throughout the body in this positron emission tomography.

Throughout imaging examinations, no space occupying lesions were detected in the liver, and the spleen appeared normal in size without evidence of enlargement. However, the onset of severe anemia, thrombocytopenia, and abnormal lymphocytes in the peripheral blood raised suspicion of hematological diseases. Bone marrow examinations subsequently confirmed lymphoma involvement in the bone marrow. Further follow-up imaging revealed a slight increase in liver volume and radioactive uptake on positron emission tomography (PET) (Fig. 6C and D). Additionally, multiple enlarged lymph nodes were identified throughout the body, including in the bilateral neck, chest, axillae, groin, abdominal cavity, and pelvic cavity. These findings, combined with the pathological examination, confirmed the diagnosis of lymphoma.

### 2.5. Pathological examination of liver

**Figure 7.**
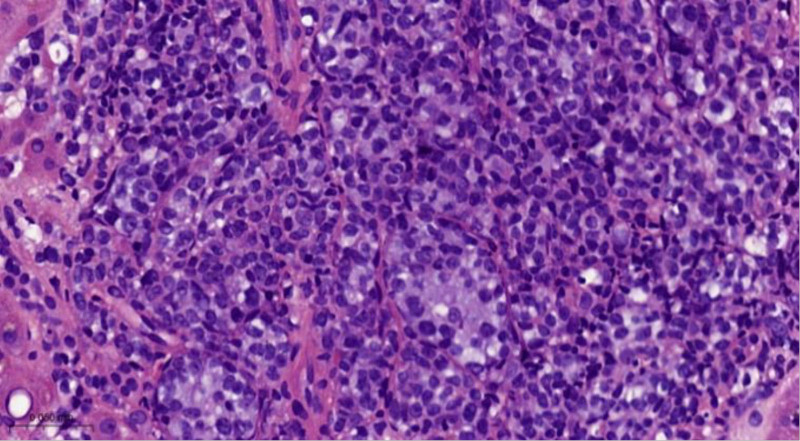
Histopathological results of liver biopsy. Hematoxylin-eosin (HE) staining, magnified by 400 times. This H&E image is representative but limited; interpretation should rely primarily on the immunohistochemical findings described in the text.

In August 2024, the patient underwent an ultrasound-guided liver biopsy under local anesthesia. Pathological examination revealed 2 samples of liver tissue, each consisting of 2 grayish-yellow strips measuring 1.5 cm in length and 0.1 cm in diameter. Microscopically, there was evidence of abnormal lymphocyte proliferation within the hepatic sinusoids, with some areas showing focal aggregation and the presence of mitotic figures (Fig. 7).

Immunohistochemical analysis demonstrated that the cells were negative for CD3, CD5, and CD10, but positive for CD20 and CD79a, with a Ki-67 labeling index of approximately 80%. The cells also expressed BCL-2, BCL-6, MUM1, and c-Myc. Molecular pathology testing confirmed that the sample was negative for Epstein-Barr virus.

### 2.6. Molecular genetic examination

Due to the limited amount of liver biopsy tissue, all molecular genetic examinations were conducted on the bone marrow samples. Cytogenetic analysis revealed a complex karyotype, with fluorescence in situ hybridization (FISH) confirming MYC and BCL-6 gene breakage. Next-generation sequencing (NGS) identified multiple gene mutations, including NOTCH2 (p.Q2361X), EP300 (p.Q1256X), KMT2C (p.Q318Sfs35), SPEN (p.Q840X), TMSB4X (p.E22Dfs14), and IRF4 (p.S114R).

Fluorescence in situ hybridization (FISH) analysis confirmed gene rearrangements involving MYC and BCL-6. Although BCL-2 gene rearrangement testing was not performed due to limited tissue availability, karyotype analysis showed no evidence of BCL-2 abnormalities. Therefore, this lymphoma does not meet criteria for a triple-hit lymphoma, and is classified as DHL.

### 2.7. Clinical diagnosis and treatment

Based on these findings, the patient was diagnosed with double-hit diffuse large B-cell lymphoma (DH-DLBCL), nongerminal center type, involving the lymph nodes, liver, spleen, and bone marrow, with an (IPI) score of 5, placing her in the very-high-risk group. This diagnosis was supported by the presence of abnormal lymphocytes, immunohistochemical evidence of B-cell markers (CD20, BCL-2, BCL-6), and the absence of T-cell markers (CD3, CD5). The Ki-67 labeling index of 80% indicated a high proliferative activity of the tumor cells.

The lymphoma diffusely involved 2 extranodal organs and was at an advanced stage with a very high IPI score. Following a multidisciplinary discussion, the decision was made to initially administer low-dose cytotoxic drugs to reduce the tumor burden before initiating the Pola-R-CHP regimen. However, prior to the administration of 1 mg vindesine, 0.2 g cyclophosphamide, and 30 mg prednisone, the patient developed persistent epistaxis, followed by melena. These symptoms were likely attributed to thrombocytopenia and coagulopathy, secondary to bone marrow and liver involvement and hepatic function failure.

Treatment included platelet transfusion, fluid resuscitation, coagulation factor supplementation, and fibrinogen administration, along with the restriction of oral intake. On the morning of July 23, 2024, the patient experienced severe symptoms, including wheezing, a significant drop in blood pressure, and the vomiting of approximately 500 mL of Black gastric contents. Emergency fluid resuscitation and symptomatic treatment were promptly initiated. Despite these efforts, the patient’s blood pressure continued to decline, and resuscitation attempts were unsuccessful. The patient was pronounced clinically dead at 7:39 am on July 23, 2024.

## 3. Discussion

Although hepatic involvement in lymphoma is common, typically occurring in approximately 50% of non-Hodgkin lymphoma cases, our patient’s clinical course was particularly unusual due to initial dominant hepatic presentation manifesting as cholestatic hepatitis without clearly identifiable liver lesions on early imaging, thus complicating early diagnosis. The delayed identification of lymphoma was partly due to the atypical presentation and nonspecific early clinical signs, including cytopenias initially attributed to other causes such as infection or inflammation.

Compared to primary hepatic lymphoma (PHL), SHL is more commonly encountered in clinical practice and can be found in nearly half of non-Hodgkin lymphoma patients. Hepatic lymphoma presents with a wide range of imaging findings, which are typically evaluated through (CT) or MRI. It may appear as a solitary mass, multiple lesions, or even a miliary pattern characterized by numerous small, discrete nodules.^[2]^ Other presentations include diffuse infiltration (with or without hepatomegaly) or hilar masses.^[2]^ In rarer cases, SHL may lack distinct imaging features.^[2]^ In this patient, diffuse infiltration was confirmed via PET/CT and liver biopsy, yet no clear imaging pattern was observed on CT or MRI.

Patients with hepatic lymphoma typically present with systemic symptoms such as fever, night sweats, and weight loss.^[3]^ Hepatosplenomegaly and generalized lymphadenopathy are commonly observed during systemic examination^3^. Tumor infiltration or extrahepatic bile duct obstruction may result in mild elevations of serum transaminases and moderate increases in alkaline phosphatase (ALP).^[3]^ It is generally accepted that SHL can often be diagnosed through imaging, as it frequently involves lymphadenopathy.^[4]^ However, in the case we report, early diagnosis was particularly challenging. The patient initially presented with abdominal pain, and imaging examinations revealed thickening of the gallbladder wall, along with mild elevations in direct bilirubin and transaminase levels. This led to the assumption that the abdominal pain was caused by an episode of cholecystitis. Poor appetite and weight loss, likely secondary to the abdominal pain, did not initially raise clinical suspicion. After several months of conservative treatments, including anti-infection measures, the patient’s abdominal pain worsened and was accompanied by fever. Repeated abdominal imaging evaluations failed to reveal significant abnormalities. It was not until the patient exhibited abnormal blood parameters – manifested as anemia, thrombocytopenia, and an increased proportion of monocytes – alongside persistent fever and weight loss, that the possibility of a hematopoietic system tumor was considered. Since the absolute value and proportion of monocytes had been elevated for over 3 months, chronic myelomonocytic leukemia was initially suspected. However, peripheral blood smears did not confirm an increase in monocytes. Instead, they revealed a significant number of abnormal lymphocytes. Due to their varying sizes and abundant cytoplasm, the testing instruments misidentified larger abnormal lymphocytes as monocytes during routine blood tests. This discovery of abnormal lymphocytes became a critical breakthrough in establishing the diagnosis. Given the presence of B symptoms such as fever and weight loss, a lymphohematopoietic system tumor was strongly suspected. Bone marrow examinations confirmed bone marrow involvement by diffuse large B-cell lymphoma (DLBCL). PET-CT imaging showed increased diffuse uptake in the liver and newly developed multiple enlarged lymph nodes with increased uptake. A liver biopsy further confirmed liver involvement by DLBCL. In this case, no abnormal blood parameters or characteristic imaging findings were detected until the disease had extensively affected the bone marrow, lymph nodes, and liver. This highlights the importance of early identification in patients presenting with unexplained abdominal pain, fever, and weight loss, particularly when imaging fails to provide a definitive diagnosis.

Given the patient’s longstanding history of Sjögre’s syndrome and elevated monoclonal IgM, the potential transformation from an underlying low-grade lymphoma, such as lymphoplasmacytic lymphoma or marginal zone lymphoma, warrants consideration. It is well-known that autoimmune conditions like Sjögren’s syndrome can predispose patients to low-grade B-cell lymphomas, which can later undergo high-grade transformation into aggressive DLBCL.^[5]^ Therefore, early microscopic examination of peripheral blood is crucial whenever cytopenias or unexplained blood abnormalities occur, as it could facilitate earlier recognition of atypical lymphoid cells and lymphoma diagnosis.

Moreover, leukemic presentation in DLBCL, although rare, usually associates with aggressive genetic aberrations, notably MYC rearrangements or double-hit/triple-hit lymphoma phenotypes. The aggressive molecular profile observed in our patient aligns well with previously reported leukemic DLBCL cases that commonly feature MYC and BCL-6 rearrangements, emphasizing the significance of prompt and comprehensive genetic evaluation in such atypical presentations.^[6,7]^

The prognosis of this patient is extremely poor, not only due to the presence of diffuse liver involvement and a high (IPI) score but also due to additional adverse prognostic factors. The presence of M-protein has been significantly associated with very poor overall survival (OS) and progression-free survival (PFS) in patients with extranodal diffuse large B-cell lymphoma (DLBCL).^[8]^ Clinical data from Chinese patients diagnosed with DLBCL revealed that chromosome abnormalities were detected in 31 of 88 patients (35.2%), with 15 of these patients (17.0%) showing a complex karyotype. The expression rates of BCL-2, BCL-6, C-MYC, and Ki-67 ≥ 80% were particularly high in patients with complex karyotypes, most of whom were double-expressor lymphomas. Survival analysis indicated that patients with a complex karyotype had poorer PFS and OS compared to those with normal karyotypes or 1 to 2 chromosomal abnormalities.^[9]^ This patient not only harbored a complex karyotype but was also diagnosed with double-hit lymphoma (DHL), a subtype of DLBCL. DHLs, characterized by rearrangements in MYC and BCL-2 and/or BCL-6, are unequivocally associated with poor outcomes following standard chemoimmunotherapy.^[10]^ In our case, molecular analysis identified rearrangements involving MYC and BCL-6 but notably lacked a rearrangement of BCL-2. Although a complete triple-hit evaluation could not be conducted, available cytogenetic data support classification as a DHL rather than triple-hit. This distinction has important prognostic implications, as triple-hit lymphomas generally confer an even worse prognosis compared to DHLs. Next-generation sequencing (NGS) in this patient revealed multiple gene mutations. Mutations in NOTCH2 allow escape from ubiquitin-dependent degradation, promoting chemoresistance in DLBCL.^[11]^ Mutations in CREBBP/EP300 induce H3K27 deacetylation, which negatively affects the NOTCH signaling pathway by dysregulating the NOTCH repressor FBXW7. This activates the NOTCH pathway, closely associated with B-cell malignancies, contributing to tumor progression in DLBCL.^[12]^ The KMT2C gene, part of the histone methyltransferase family, catalyzes methylation of histone H3 lysine 4 (H3K4), a modification linked to gene activation. Mutations in KMT2C disrupt this function, leading to abnormal H3K4 methylation levels, dysregulated gene expression, and malignant transformation of tumor cells. These mutations are thought to contribute to lymphoma invasiveness and poor prognosis.^[13]^ Molecular classifications of DLBCL have identified the BN2 subtype as having relatively favorable prognoses due to activation of the BCL-6 and NOTCH signaling pathways.^[14]^ However, the truncating/inactivating mutation of SPEN, observed in this patient, may indicate high-risk molecular features. SPEN normally regulates the NOTCH signaling pathway by forming repressive complexes that inhibit NOTCH target genes. Studies have shown that SPEN mutations are enriched in DLBCL cases failing to achieve event-free survival at 24 months (EFS24), contrasting with generally favorable outcomes in BN2 subtype tumors.^[15]^ The TMSB4X protein is involved in regulating the intracellular actin cytoskeleton, crucial for cell migration, proliferation, and differentiation. Mutations in TMSB4X may enhance the migration and invasion potential of tumor cells.^[16]^ IRF4, a key transcription factor in B-cell differentiation, immune regulation, and metabolism, has mutations closely associated with poor lymphoma prognosis.^[17]^ This combination of genomic instability and multiple gene mutations likely contributed to the patient’s rapidly progressing disease.

Plasmablastic lymphoma (PBL) is an aggressive subtype of diffuse large B-cell lymphoma typically characterized by immunoblast-like or plasmacytoid tumor cells with eccentrically located nuclei, prominent nucleoli, and abundant basophilic cytoplasm. Immunophenotypically, PBL generally lacks or weakly expresses conventional B-cell markers such as CD20 and PAX5 but strongly expresses plasma cell markers including CD38, CD138, and MUM1/IRF4. Additionally, PBL frequently occurs in the context of HIV infection or other immunocompromised states and commonly harbors MYC translocations, contributing to its aggressive clinical behavior.^[18,19]^

In contrast, our case displayed strong positivity for pan-B cell markers, notably CD20 and CD79a, while CD138 – a characteristic plasma cell marker for PBL – was negative. Moreover, despite showing MUM1 positivity, our patient had no MYC translocation as would typically be seen in PBL. Clinically, the patient was immunocompetent and HIV-negative, further reducing suspicion for PBL. Thus, based on both immunophenotype and clinical context, our case is best classified as a double-hit DLBCL with atypical hepatic involvement, distinctly different from plasmablastic lymphoma.

In terms of treatment, the most commonly used immunochemotherapy regimen for diffuse large B-cell lymphoma (DLBCL) is R-CHOP, which includes rituximab, cyclophosphamide, doxorubicin, vincristine, and prednisolone.^[20]^ In patients with previously untreated intermediate-risk or high-risk DLBCL, the risk of disease progression, relapse, or death was significantly lower among those treated with pola-R-CHP (polatuzumab vedotin with R-CHP) compared to those receiving R-CHOP.^[21]^ For patients with (PHL), a combination of surgical resection and chemotherapy has demonstrated improved outcomes.^[22]^ However, surgical intervention is generally not a viable option for patients with SHL.

Before the initiation of formal first-line treatment, the patient was administered low-dose cytotoxic drugs to reduce the tumor burden. During this period, she experienced epistaxis and melena. The hemorrhagic events were likely caused by thrombocytopenia and coagulopathy. Bone marrow involvement by lymphoma led to megakaryocyte dysfunction, while liver involvement resulted in reduced thrombopoietin (TPO) production, both contributing to severe thrombocytopenia. Additionally, hepatic involvement impaired the liver’s ability to synthesize coagulation factors, further exacerbating coagulopathy. Given the low dose of cytotoxic drugs, myelosuppression from chemotherapy was not considered a contributing factor. Subsequently, the patient’s blood pressure dropped suddenly, which was initially attributed to increased gastrointestinal hemorrhage. However, the possibility of intra-abdominal hemorrhage caused by liver tumor rupture could not be excluded. Although rare, both primary and secondary hepatic lymphoma can result in liver tumor rupture, leading to life-threatening intra-abdominal hemorrhage.^[23]^ The most common symptom of tumor rupture is the sudden onset of abdominal pain accompanied by shock. In this case, the patient was unable to verbally express whether she experienced a sudden exacerbation of abdominal pain but did present with a sudden drop in blood pressure and signs of shock. While gastrointestinal hemorrhage was the most likely cause, the possibility of liver tumor rupture and intra-abdominal hemorrhage also needed to be considered.

## 4. Conclusion

Compared to (PHL), SHL is more common, and most cases can be diagnosed through clinical symptoms, laboratory tests, and imaging studies. However, we report an atypical case of SHL, which presents new challenges for diagnosis. While advancements in imaging techniques can aid in diagnosis, definitive confirmation still relies on histopathological evaluation. The R-CHOP and Pola-R-CHP regimen remains the gold standard for treatment. Further research is needed to enhance diagnostic capabilities and improve treatment efficacy.

## Author contributions

**Conceptualization:** Yanying Wang.

**Data curation:** Dongliang Zhao.

**Funding acquisition:** Yanying Wang.

**Methodology:** Dongliang Zhao.

**Project administration:** Dongliang Zhao.

**Resources:** Lihong Li.

**Visualization:** Lihong Li.

**Writing – original draft:** Dongliang Zhao.

**Writing – review & editing:** Dongliang Zhao, Lai Wei, Yanying Wang.
